# Hydrochar as a One Health solution to mitigate antibiotic resistance genes from slurry in grassland soils

**DOI:** 10.3389/fmicb.2026.1800404

**Published:** 2026-05-13

**Authors:** Israel Ikoyi, Selva Dhandapani, Rory Doherty, Sadish Oumabady

**Affiliations:** 1 School of Biological, Earth and Environmental Sciences, & Sustainability Institute, University College Cork, Cork, Ireland; 2 Soil Biogeochemistry and Terrestrial Ecology Research Programme, Agri-Food Bioscience Institute (AFBI), Belfast, United Kingdom; 3 School of Natural and Built Environment, Queen's University, Belfast, United Kingdom

**Keywords:** animal agriculture, grassland soils, hydrothermal carbonization, slurry management, soil health, soil microbes, sustainable agriculture

## Abstract

Antibiotic resistance genes (ARGs) represent a growing global health concern, with agricultural practices, particularly livestock slurry application, serving as major contributors to their spread in soils. This review evaluates hydrothermal carbonization derived hydrochar as a potential One Health strategy for reducing ARG prevalence in slurry amended grassland systems. We synthesize current evidence on how hydrochar's properties and interactions within slurry-soil-grassland environments can limit ARG persistence, including its capacity to bind genetic material, affect microbial populations, and alter soil conditions that influence resistance dynamics. Where direct evidence from hydrochar based studies were lacking, findings from analogous materials such as biochar in related agricultural systems were used to elucidate the likely properties and impacts of slurry-derived hydrochar when applied to grassland slurry systems. In doing so, we highlight the potential of hydrothermal carbonization technology to contribute to addressing several environmental challenges in temperate grassland systems, where slurry management represents a persistent concern. Broader ecological and practical implications, such as impacts on soil function, microbial diversity, and the sustainability of hydrochar production, are also considered. Overall, this review highlights the potential of hydrochar as a tool for limiting the dissemination of ARGs from agricultural slurry and emphasizes the need for further research to optimize its application within sustainable grassland management. By synthesizing current evidence and identifying critical knowledge gaps, we propose hydrochar as a promising One Health intervention in slurry-soil-grassland systems and outline key research priorities required to realize its full potential.

## 1 Introduction

Antimicrobial resistance (AMR) is one of the most important global challenges currently facing the world and is predicted to become the leading cause of human mortality in the twenty-first century (O'Neill, 2016; Pagaling et al., 2023). Discovery of antibiotics in the twentieth century changed the face of modern medicine, and increased the human lifespan by 23 years, which is now being threatened by development of AMR (Hutchings et al., 2019a,b; Han et al., 2022). It is estimated that by 2050, drug resistant infections due to high AMR prevalence, could cause 10 million deaths a year, and could have highly significant cumulative effect on economic output affecting human development (O'Neill, 2016). In addition to the direct use of antibiotics (antimicrobial drug) in modern medicine to treat infections, indirectly many medical procedures such as surgeries and chemotherapy will become too dangerous to perform if AMR persists (Hutchings et al., 2019a; Nanayakkara et al., 2021; Dhole et al., 2023).

AMR forms a core of One Health connections between soil, plant, animal and ecosystem health. Most of the antibiotics are natural compounds produced as secondary metabolites by microbes such as bacteria and fungi (Sengupta et al., 2013). These microorganisms live in the soil, which is home to most (~60%) of the biodiversity on earth (Anthony et al., 2023), and hence most (~80%) of the antibiotics that are used clinically are primarily sourced from soil (Han et al., 2022). Microbes produce these antibiotics to eliminate competing co-occurring microbes and plays a crucial role in plant-soil interactions in ecosystems. Interruption of this balance in competition through AMR could have impact on wider soil microbiome and affect functionally important groups that maintain soil and plant health, with further transport through food chain to animal and human microbiome (Chen Q. L. et al., 2019; Wang et al., 2022). Therefore, AMR exposure in soils poses a serious threat to One Health.

Grassland soils are particularly vulnerable to this threat of increase in AMR, due to application of slurry with high microbiological loading. Application of slurry to grassland soils is one of the easily available, cheap and organic solutions for nutrient deficiency in soil, serving also as a convenient animal waste management in farms. The scarcity of rock phosphate, in addition to rising costs of production of synthetic fertilizers have further driven the increase in the use of slurry in grasslands (Brummerloh et al., 2023). However, different farm management strategies result in different chemical additions to the slurry, especially in intensive farming systems, which when transferred to soils pose several risks to soil sustainability (Brennan et al., 2011; Owusu-Twum et al., 2017). One of the important risks is the passage of antibiotics that were used as prophylactic medicine surviving the animal gut and slurry storage, into grasslands. This makes livestock slurry a leading source for AMR due to growing use of antibiotics in livestock production (Acosta et al., 2025). This is particularly relevant to cold-temperate livestock production systems where animals are kept indoors during the winter period, with the produced slurry stored in tanks, prompting further evolution of AMR genes. Application of slurry to grasslands transfers these AMR genes to grassland soils affecting soil, animal and human health, forming a core intersection for issues regarding One Health.

In addition to antibiotics, mineral nutrition supplement used in livestock production systems could persist through the passage from feed to slurry and add heavy metals and potentially toxic elements to soil that could indirectly contribute to the development of soil AMR (Pan et al., 2023; Gourlez et al., 2024). Elements like copper, cadmium and zinc are known to have antimicrobial properties and are among the most common heavy metal contaminants in soils (Angon et al., 2024). Increased availability of these elements can create AMR in microbial communities that have evolved in such environments, due to development of co- or cross-resistance (Song et al., 2017; Pan et al., 2023). There is strong evidence from a long-term experiment investigating slurry application on grassland soil, that indicate accumulation of zinc and copper in soils with increased rate of slurry applications (De Conti et al., 2016). Similarly, other studies have shown increased bioavailability of these potentially toxic elements and heavy metals with slurry application.

The management of livestock slurry has become a major environmental and public health concern due to its high load of nutrients, organic matter, antimicrobials, and antibiotic resistance genes (ARGs). Conventional treatments such as aerobic composting, anaerobic digestion (AD), and, to a lesser extent, advanced chemical treatments (e.g., liming, advanced oxidation, ammonia treatment) were used to stabilize the slurry and reduce pathogen and contaminant loads (He et al., 2020). Previous studies have shown that composting and AD can substantially reduce antibiotic resistant bacteria and partially reduce ARGs in livestock manure and slurry (Katada et al., 2021; Zhao et al., 2025). For instance, studies on dairy manure application report a strong suppression of antibiotic-resistant bacterial communities (around 1,000-fold reduction) under both composting and mesophilic AD processes. Composting often has limited or gene specific effects on ARG copy numbers, while AD typically achieves about 1 log reductions of several resistance genes, with ARGs still detectable in the digestate. This suggests both composting and AD only reduce to a less extent but do not eliminate ARGs and the outcomes might vary with temperature (mesophilic vs. thermophilic), retention time, and system design (Katada et al., 2021). Although chemical and physico-chemical treatments further inactivate microorganisms and degrade antimicrobials, they are often energy intensive, costly, and less practical for on farm use at large scale. In addition, the presence of residual antibiotics after integrated treatment (chemical and biological) correlates positively with the antibiotic gene abundance, thus building up the risk of slurry application to the agricultural fields. Along with the GHG emission and nutrient loss, these limitations underscore the need for additional or complementary slurry treatment technologies that are both cost effective and environmentally sustainable (Yan et al., 2024).

Hydrochar, a carbonized material generated from one of the thermochemical biomass conversion technologies, has emerged as a promising alternative or supplementary strategy for managing wet carbon rich (>30%) slurry and mitigating ARG dissemination. The process known as hydrothermal carbonization (HTC) or wet torrefaction involves treating the wet biomass at 180–250 °C for 5–240 min under autogenerated pressure conditions, converting it into a carbon rich solid (hydrochar), an aqueous phase, and a negligible gaseous fraction, primarily CO_2_ (Oumabady et al., 2023). Unlike conventional pyrolysis, HTC can process feedstocks with moisture contents above 80–90% (typical of animal slurry) without prior energy intensive drying, transforming it from a complex time-consuming process into a rapid simple process to produce energy efficient carbon product (Hejna et al., 2022).

During HTC, the organic components of slurry undergo a series of chemical reactions such as dehydration, decarboxylation, depolymerization, and aromatization, yielding condensed micro or nano-spherical carbon structures (hydrochar). Experimental studies indicate that HTC conditions can effectively inactivate pathogens and degrade thermally labile contaminants, while substantially transforming the matrix that hosts ARGs, thereby offering potential for ARG risk reduction for downstream applications. Hydrochar derived from biomass and manures have been proven to exhibit favorable characteristics as an alternative solid fuel, adsorbent for contaminants and pollutants from wastewater (activated carbon precursor), with recent research focusing more on its potential applications in soil amelioration and carbon sequestration (Vu et al., 2025).

**Figure 1:**
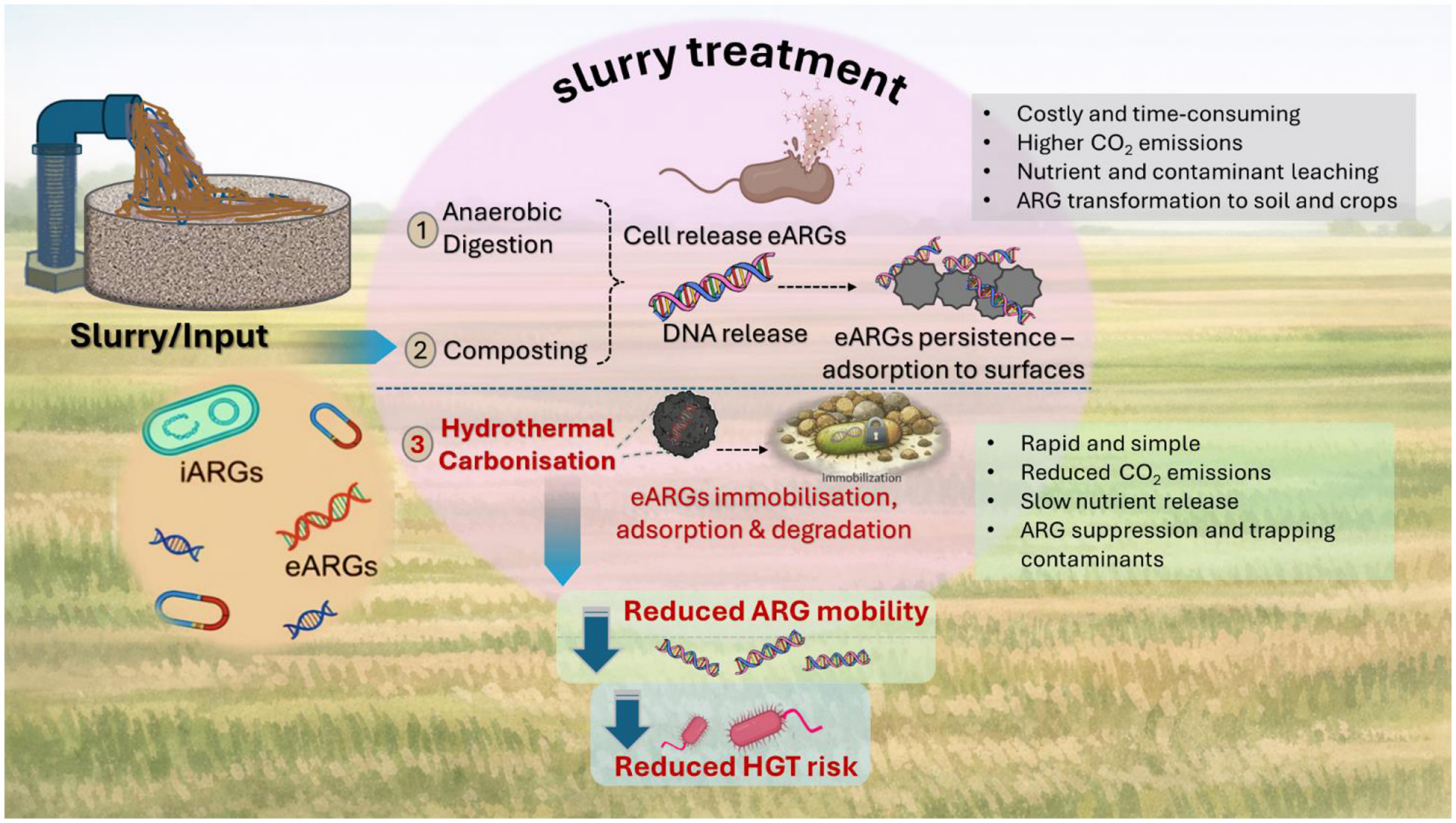
A brief graphical representation of existing and proposed slurry valorization to prevent ARGs effect in soil.

Hydrothermal treatment of biomass can concentrate carbon in a stable form while reducing the bioavailability of certain pollutants; however, largescale and long-term studies on how hydrochar from slurry affects ARG abundance and mobility in soils are still limited. Compared with composting and AD, HTC operates at higher temperatures, shorter reaction times, and does not require inert gas atmosphere, which can simplify operation and enhance treatment robustness for high moisture slurries (Chi et al., 2025). Integrating HTC and hydrochar utilization into slurry management systems represents a promising avenue to complement existing biological treatments, promote waste valorization and circular economy, and help interrupt environmental pathways of ARG dissemination (Figure 1).

The objectives of this review are

° Synthesize current knowledge on hydrochar's effects on ARGs in slurry.
° Investigate the mechanisms of ARG reduction by hydrochar in slurry-soil systems.
° Evaluate the potential of hydrochar for sustainable slurry management and ARG mitigation in grassland ecosystems.
° Identify research needs and future directions.

## 2 ARG occurrence, dissemination, and persistence in slurry-grassland systems

### 2.1 Occurrence of ARGs in slurry and grassland soils

The land application of animal slurry to grassland soils is a foundational agricultural practice globally, serving as a critical mechanism for nutrient recycling and cost-effective waste management. However, slurry is a complex biological and chemical matrix that contains not only essential macronutrients but also antibiotic residues, antibiotic-resistant bacteria (ARB), and a suite of ARGs (Wang et al., 2015). Consequently, the frequent application of slurry to agroecosystems establishes a significant environmental pathway for the dissemination of AMR, potentially facilitating the transfer of resistance determinants from the soil reservoir to the human food chain and groundwater systems (Zhu et al., 2017).

Grassland ecosystems possess unique structural and microbial characteristics that distinguish the fate of ARGs from that of intensive arable systems. Permanent grasslands are characterized by minimal soil disturbance, extensive fungal networks, and highly stratified vertical profiles. The absence of annual tillage, coupled with the cumulative impact of repeated slurry applications and grazing pressure, creates a distinct selective environment that may either buffer against or facilitate the long-term persistence of exogenous ARGs (Chen C. et al., 2019).

Livestock slurry, particularly from bovine and porcine operations acts as a primary reservoir for ARGs. The dairy cattle resistome is frequently dominated by macrolide-lincosamide-streptogramin (MLS) and tetracycline resistance genes, reflecting the historical and ongoing use of these classes in veterinary therapeutics (Wichmann et al., 2014). Recent meta-analyses indicate that cattle manure typically exhibits significantly higher ARG concentrations compared to municipal compost, with sulfonamides (sul) and tetracyclines (tet) identified as the most prevalent contaminants in manure-amended soils (Zhang et al., 2022).

In contrast, indigenous grassland soils without recent exposure to organic fertilizers harbor a “background” resistome shaped by natural evolutionary pressures. These native communities are often dominated by multidrug, bacitracin, and rifamycin resistance genes (Forsberg et al., 2014). While slurry application introduces exogenous ARGs, the long-term composition of the soil resistome is governed by a complex interplay between the introduced “invaders” and the established “native” microbial community structure.

### 2.2 Mechanisms of dissemination: horizontal gene transfer and co-selection

The environmental dissemination of ARGs is driven primarily by horizontal gene transfer (HGT) rather than the simple vertical proliferation of introduced host bacteria (Bengtsson-Palme et al., 2018). Class 1 integrons (intI1) serve as pivotal vectors in this process; a strong positive correlation between intI1 and sul1 abundances in amended soils suggests that HGT facilitates the spread of resistance among indigenous soil bacteria (Gillings et al., 2015).

Furthermore, the persistence of ARGs is frequently exacerbated by co-selection pressures. Heavy metals typically present in slurry, such as copper (Cu) and zinc (Zn), can trigger cross-resistance or co-resistance mechanisms, where the same genetic element or linked elements confer resistance to both metals and antibiotics (Pal et al., 2015). Abiotic factors, including soil pH, organic carbon content, and mean annual precipitation, have been shown to explain up to 30% of the variation in ARG abundance, highlighting the role of environmental context in shaping resistance dynamics (Yang et al., 2021).

### 2.3 Persistence dynamics and cumulative effects

Temporal studies suggest that while a single slurry application leads to a transient “spike” in ARG relative abundance (often returning to baseline within 60–90 days), repeated applications may alter the baseline equilibrium of the soil resistome (Jechalke et al., 2013). This “cumulative effect” is modulated by application frequency; frequent, low-dose applications have been shown to maintain higher ARG densities compared to infrequent, high-dose events (Chen C. et al., 2019).

Soil depth also plays a critical role in persistence. Subsurface layers (30–90 cm) often exhibit lower microbial competition and reduced UV exposure, potentially leading to longer ARG half-lives compared to surface soils (Zhang et al., 2022). Biotic resistance from the indigenous microbiome remains the primary defense against ARG invasion, yet this resilience can be compromised by environmental stressors or excessive nutrient loading.

## 3 Hydrochar production and properties relevant to slurry treatment

### 3.1 Hydrothermal carbonization (HTC) of slurry

During the HTC of slurry under subcritical water conditions, a series of chemical reactions take place that include hydrolysis, dehydration, decarboxylation, and polymerization reactions. These reactions convert the organic fraction of slurry into hydrochar, an aqueous phase enriched in dissolved organics and nutrients, and a minor gaseous fraction. Studies over the past decade on sewage sludge, dairy waste, and anaerobic digestate have shown that process parameters especially temperature, residence time, and water-to-biomass ratio strongly influence hydrochar yield, energy densification, surface chemistry, and suitability for downstream environmental applications (Ha et al., 2024; Malhotra et al., 2025).

#### 3.1.1 Temperature

Temperature is a dominant factor governing reaction pathways and product distribution during HTC of slurry and other wet biomasses. At lower temperatures within the HTC range (around 180–200 °C), ionic reactions and hydrolysis prevail, promoting solubilization and partial depolymerization, whereas at higher temperatures (220–250 °C) homolytic bond cleavage and more extensive decarboxylation increase gas formation and reduce solid yield. Consistent with earlier HTC studies and a recent work on HTC optimization in sludge confirmed higher processing temperatures generally decrease hydrochar mass yield while increasing carbonization degree, aromaticity, and the proportion of gaseous products (Portilla-Amaguaña et al., 2024). Proximate and elemental analyses reported for HTC of manures, sewage sludge, dairy processing waste, and green harvesting residues show that increasing temperature leads to higher fixed carbon content, ash content and lower volatile matter in the resulting hydrochar, accompanied by increased calorific value and greater hydrophobicity. For slurry derived hydrochars, these trends are advantageous for fuel and carbon sequestration applications but must be balanced against lower solid yields when the goal is maximum carbon recovery from slurry (Wilk et al., 2023).

#### 3.1.2 Residence time

Residence time strongly influences the distribution and quality of solid, liquid, and gas proportions, as well as the internal structure of hydrochar. Longer residence times increase reaction severity, enhancing degradation of dissolved and particulate organics, but typically reduce hydrochar yield as more carbon is transferred to the aqueous and gaseous phases. Studies on HTC of lignocellulosic residues, sewage sludge, and waste-activated sludge indicate that extended residence times (>4 h) promote further condensation and polymerization of dissolved intermediates, forming secondary hydrochar with enhanced polyaromatic structures and a higher proportion of stable oxygen-containing functional groups (Shahnawazi et al., 2025). Microscopic and spectroscopic analyses have shown that residence time also affects hydrochar morphology: shorter reaction times (≤6 h) often yield particles with relatively smooth surfaces, limited microsphere formation, and small cracks, whereas longer times (>6 h) can lead to pronounced carbon microsphere formation and more developed porous structures. For slurry applications, tuning residence time therefore allows adjustment of surface area, porosity, and functional group distribution, which are critical for hydrochar's performance as an adsorbent or soil amendment while also influencing its stability and potential interactions with antibiotics and ARGs (Ha et al., 2024). Hydrochar properties like surface area, pore volume and oxygen-to-carbon ratio have proven to reduce the bioavailability of persistent antibiotics in soil such as Oxytetracycline (Lang Q. et al., 2023).

#### 3.1.3 Water-to-biomass ratio

An adequate water-to-biomass ratio is essential to ensure complete dispersion of solids and efficient heat and mass transfer during HTC. Several works on coniferous biomass, agricultural residues, green harvesting residues, and sewage sludge shows that higher water-to-biomass ratios intensify hydrolysis and solubilization of organics, often leading to slightly reduced solid yields but improved homogeneity of treatment and more efficient leaching of inorganics. In contrast, lower ratios favor condensation and carbonization, frequently increasing hydrochar carbon content and hydrophobicity at the expense of process uniformity that sometimes results in incomplete hydrolysis of highly concentrated slurries (Merzari et al., 2020).

For sewage sludge and manure-like feedstocks, experiments have demonstrated that increasing the water-to-biomass ratio within typical HTC ranges (9:1 to 10:1) can increase the apparent hydrochar solid yield but simultaneously modify proximate and elemental composition. In some cases, lower water-to-biomass ratios enhanced fixed carbon content relative to the raw substrate, while higher ratios produced larger solid yields with lower fixed carbon content, highlighting that both yield and quality respond non-linearly to dilution. The optimal water-to-slurry ratio for HTC depends on the intended end use of slurry-derived hydrochar (Khalaf et al., 2022).

### 3.2 Feedstock variability

Slurry from different livestock and poultry display markedly different dry matter, volatile solids and ash contents, available nutrients (N, P, K), and trace metal concentrations. Dairy slurries tend to be richer in lignocellulosic fibers due to forage-based diets, whereas pig and poultry manures generally contain higher nitrogen and phosphorus concentrations and lower fiber fractions, leading to higher ash contents and different nitrogen speciation in the resulting hydrochar. Diet formulation (e.g., high concentrate vs. high forage rations, crude protein level, mineral supplementation) alters the relative contributions of structural carbohydrates, proteins, and lipids in excreta, and these compositional shifts have been linked to measurable differences in HTC mass yield, hydrochar C/N ratio, and nutrient distribution between solid and liquid phases (He et al., 2020). Studies on hydrochar's interaction with ARGs are very limited or at a developing stage. For instance, ARG interaction were studied with hydrochar made from pig slurry and spent mushroom substrate (Shan et al., 2023; Chi et al., 2025). Hydrochar derived from spent mushroom substrate exhibited a plant based, low heavy metal composition with abundant oxygen containing functional groups, leading to ARG risk reduction primarily through indirect mechanisms such as microbial community restructuring and suppression of dominant ARG host taxa during composting (Shan et al., 2023). In contrast, pig manure derived hydrochar contained higher ash and residual metal contents and achieved ARG risk reduction mainly via direct thermo-chemical destruction of ARGs and MGEs and temperature dependent immobilization of bioavailable Cu and Zn, thereby substantially inhibiting horizontal gene transfer but requiring stricter process control to mitigate metal-associated risks (Chi et al., 2025). However, it is premature to draw definitive conclusions directly linking feedstock type to hydrochar mediated ARG risks or mitigation pathways, as the available evidence is derived from two studies conducted under different experimental designs and process conditions. Broader, systematically comparable datasets across diverse feedstocks, operating parameters, and environmental settings are therefore required before robust generalizations on feedstock-driven ARG behavior can be established.

Antibiotic usage adds another layer of variability, since concentrations of tetracyclines, sulfonamides, macrolides, and their metabolites in livestock manure can vary across orders of magnitude between production systems and regions. These compounds, together with co-selective metals (e.g., Cu, Zn from feed additives), influence the speciation and mobility of contaminants in both hydrochar and HTC process water, and shape the ARGs and mobile genetic elements carried into soil (Song et al., 2017). Anaerobic digestate used as HTC feedstock differs systematically from raw slurry, typically exhibiting lower concentrations of easily degradable organics, a higher fraction of microbial biomass, and more mineralized forms of nitrogen and phosphorus (e.g., ammonium and orthophosphate), leading to distinct hydrochar ash composition and dewaterability compared with hydrochar from undigested slurry. Previous HTC studies on digestate and waste-activated sludge consistently report that initial volatile solids content, organic/inorganic ratio, and macromolecular composition (protein versus carbohydrate versus lipid dominance) strongly condition hydrochar energy density, ash fraction, and adsorption behavior (Vu et al., 2025). Protein and nitrogen rich wastes tend to generate more ammonium and basic nitrogen species in the process water, partially buffering pH and moderating the formation of organic acids, whereas carbohydrate and lignocellulose rich slurries produce higher concentrations of low molecular weight acids such as acetic, formic, and levulinic acids, which drive a stronger pH decrease and accelerate dehydration and decarboxylation reactions. In digestates and waste-activated sludge with high inorganic and ash contents, the dissolution of minerals (e.g., Ca, Mg, Fe, Al) into the liquid phase can buffer the pH drop and alter the speciation of phosphorus and metals, affecting both hydrochar ash composition and its suitability as a fuel or soil amendment (Prieto et al., 2024; Gong et al., 2025).

### 3.3 Key hydrochar properties

The critical hydrochar properties for mitigating ARGs include high surface area/porosity for antibiotic sorption, surface charge and functional groups for microbial immobilization, pH and cation exchange capacity (CEC) for ecological shifts, nutrient stabilization, and heavy metal binding to disrupt co-selection (Shan et al., 2023).

High surface area and well-developed porosity enhance sorption of antibiotics and co-selecting contaminants, lowering their bioavailability and thus reducing selective pressure that drives ARG proliferation in soil microbial communities. Raw hydrochars from slurry/digestate typically exhibit low BET surface areas (3–20 m^2^ g^−1^) but develop meso/macropores during HTC that trap ARG-hosting microbes and antibiotics (Khosravi et al., 2022). Hydrochars are found to have more chemically interactive properties of adsorbents that were found to be less in other forms of carbon-rich materials like biochar. The intermediate reactions during HTC enrich the hydrochar's surface with oxygenated functional groups such as carboxylic, ketonic, hydroxyl and phenolic groups. The combined influence of these oxygenated functional groups and surface charge of hydrochar promote electrostatic attraction, hydrogen bonding, and complexation with antibiotics, heavy metals, and mobile genetic elements, which can suppress horizontal gene transfer. The conversion of digestate of swine manure and rice straw to hydrochar at 190 °C with a ratio of biomass and water at 1:4 and 1:9 synthesized hydrochars with acidic pH and better BET surface area, more oxygenated functional groups, and aromatic C=C and C-H band were present as compared to the original feedstock and pyrochars (Jin et al., 2017).

The intrinsic pH and associated cation exchange capacity (CEC) of hydrochar produced from different feeds influence soil pH buffering and ion exchange processes. This indirectly shapes microbial community structure and ARG profiles by modifying habitat conditions and nutrient retention. Nutrient-rich hydrochars can promote selective stimulation of microbial activity and biomass, potentially diluting ARG-carrying populations (Shan et al., 2023). Heavy metal content and speciation are critical because metals can act as co-selective agents for ARGs; hydrochars that immobilize metals through complexation and precipitation are more favorable than those that introduce readily bioavailable metals, which may enhance co-selection of resistance (Khosravi et al., 2022).

Hydrochar application has been proven to improve the physical properties of soil in terms of porosity, bulk density, water holding capacity and available water capacity. The structural morphology of hydrochar denotes a meso/macro-porous surface which makes it a localized habitat for the soil microbes, specifically the fungal communities (arbuscular mycorrhizal fungi). It also helps in promoting the activity of microbes involved in decomposition of labile organic carbon fractions and stabilizes the soil organic carbon pool by improving the proportion of aromatic compounds thereby ensuring the carbon sequestration potential (Oumabady et al., 2023). Gaseous emissions from soil could also be influenced by hydrochar application, for instance, reduction in volatilization of ammonia and improving the fertilizer use efficiency of urea. The nutrients embedded in the structural skeleton of hydrochar promotes nutrient supply to plants consistently. The acidic nature of hydrochar makes it preferable for the reclamation of saline alkali soils. The oxygenated functional groups such as carboxylic, phenolic OH, and ketonic groups were found to be higher in the hydrochar thereby making it as a reactive component and improves the cation CEC of the soil (Oumabady et al., 2023). This enhanced CEC will retain cations reducing bioavailability of PTEs (e.g. Cu & Zn which are ARG promoting as well as other PTEs reducing contaminant transport in soils, surface water and groundwater). Hydrothermal carbonization is a promising approach of biosolids management and its utilization as a soil amendment. The physical and chemical properties of hydrothermally converted biosolids and its effect as a potential soil amendment on the growth of agricultural crops are currently being studied around some of the global institutions.

### 3.4 Comparison between hydrochar and biochar

Compared to hydrochar production from hydrothermal carbonization, biochar is produced by pyrolysis of biomass in the absence of oxygen at temperature range of 400–500 °C. From the previous work of Oumabady and colleagues, a simple cost workout of biochar and hydrochar production is presented in Table 1 (Oumabady et al., 2023). Pyrolysis facilitates the removal of moisture, volatile compounds and other aromatics with pore formation (Kambo and Dutta, 2015). Carbonization have a substantial effect on carbon production which encompasses careful selection of the paralyzing factors. The carbonization temperature has the most significant effect, followed by the heating rate, presence or absence of an inert atmosphere and its rate, and depends finally on residence time in this process.

**Table 1:** Comparisons between hydrochar and biochar on several production parameters.

Parameters	Hydrochar	Biochar
Type of waste	Wet waste (≥65%)	Dry waste (≤30%)
Operating temperature	180–250 °C	600–800 °C
Pressure requirement	15–30 bar	1 bar
Installation capacity (kg)	250–300	150–200
Processing time (hour)	3-4	1–2
Waste processed per day (kg)	500–800	800–1,000
Char produced per day (kg)	120–150	300–400
Capital cost (£)	20,000–25,000	15,000–20,000
Operational cost per day (£)	50–60	50–60

Wastes with distinct carbon and moisture content can be utilized to produce carbonaceous materials with further applications in energy production, wastewater treatment and soil amelioration. Wastes with lower moisture content can be pyrolyzed to produce biochar while wastes with higher moisture content can be hydrothermally carbonized to produce hydrochar. These carbonized products have been proven to be worthy eco-friendly materials for utilization as energy alternatives to coal, adsorbing materials for wastewater treatment and as soil amendments (Takaya et al., 2016; Bardhan et al., 2021; Suarez et al., 2023). Based on comprehensive literature, we have identified a generalized comparison between hydrochar and biochar on ARGs reduction as listed in Table 2 (Shao et al., 2022; Wu Y. et al., 2022; Wu C. et al., 2022; Wu et al., 2025; Shan et al., 2023; Jia et al., 2024).

**Table 2:** Comparison between hydrochar and biochar in ARG removal.

Characteristics	Hydrochar	Biochar
Surface properties	Comparatively lower surface area, more oxygen containing functional groups	High surface area, porous and aromatic carbon matrix
Dominant removal mechanisms for ARGs	Microbial regulation, pathogen suppression	Electrostatic/π-π interactions
Effect on ARG abundance	Significant reduction of mobile genetic elements	Promote degradation based on the matrix (soil, sludge or wastewater)
Mechanistic pathways	Alters microbial community structure and metabolism (carbohydrate/protein)	Adsorption, oxidative degradation
Modification potential	Less explored	Metal doped/activated biochar improves ARG removal

## 4 Mechanisms of ARG reduction by hydrochar in slurry-soil systems

### 4.1 Direct interactions with ARGs and bacteria

Beyond altering the selective pressures in the environment, hydrochar can exert direct effects on ARGs and the bacteria that carry them. These effects, which include adsorption, growth inhibition, and physical disruption, can significantly influence the persistence and dissemination of antimicrobial resistance in treated matrices such as soil, water, and manure. While these direct effects have been well-documented for biochar (Jang and Kan, 2022; Sun et al., 2023; Shan et al., 2024), very few studies are available on hydrochar. Given the unique characteristics of hydrochar, we propose this as a potential area of research to explore.

#### 4.1.1 Adsorption of ARGs and antibiotics onto hydrochar surfaces

A primary mechanism by which hydrochar directly impacts ARG fate is through the adsorption of extracellular ARGs (eARGs) and co-occurring antibiotics onto its surface. The persistence of eARGs in the environment is a critical concern, as they can be taken up by competent bacteria via horizontal gene transfer, even after the originating cells have died (Zhang et al., 2020a; Kittredge et al., 2022). Hydrochar, with its specific surface properties, can act as a potent sorbent. While typically possessing a lower specific surface area than biochar, hydrochar has a greater abundance of oxygen-containing functional groups (e.g., hydroxyl, carboxyl) on its surface, which facilitate interactions with biomolecules (Nguyen et al., 2021). Studies have demonstrated that hydrochar can effectively adsorb ARGs, through mechanisms such as electrostatic interactions and hydrogen bonding (Ngoc et al., 2023). This sequestration can reduce the bioavailability of eARGs for transformation, thereby potentially reducing the spread of horizontal gene transfer.

Furthermore, the concurrent adsorption of antibiotic residues onto hydrochar presents a synergistic effect. By removing antibiotic selective pressure from the immediate environment, hydrochar reduces the competitive advantage of ARG-carrying bacteria (Shan et al., 2023). However, the loaded hydrochar becomes a point of concentrated antibiotics and ARGs, and the long-term stability of this adsorption and the potential for subsequent desorption under changing environmental conditions requires further research.

#### 4.1.2 Inhibition of bacterial growth due to the presence of inhibitory compounds in hydrochar

The hydrothermal carbonization process does not only carbonize biomass; it also generates a complex cocktail of organic compounds, some of which possess microbial toxicity (Usman et al., 2023). These compounds, including phenols and organic acids, can be either deposited on the hydrochar surface or released into the surrounding aqueous phase as leachates (Hao et al., 2018). The presence of these inhibitory substances can directly suppress the growth and metabolic activity of a broad spectrum of bacteria, including those hosting ARGs.

For instance, phenolic compounds, known for their antimicrobial properties, can penetrate cell membranes and cause protein denaturation and enzyme inhibition (Oulahal and Degraeve, 2022). This non-selective toxicity can reduce the overall bacterial abundance, including ARG hosts, thereby lowering the absolute number of potential vectors for ARG dissemination. Research by Campagnano et al. (2024) showed inhibition in the growth of *Escherichia coli and Bacillus subtili* when exposed to both hydrothermal carbonization (HTC) leachates and hydrochars, correlating with high salinity, high nutrients, dissolved organic carbon, and other ions in the leachate. It is crucial to note that this inhibitory effect is highly dependent on hydrochar feedstock and process conditions, as these factors dictate the type and concentration of by-products generated (Shanmugam et al., 2025).

#### 4.1.3 Disruption of bacterial cell membranes by hydrochar's physical and chemical properties

Although direct experimental evidence specifically showing membrane rupture by hydrochar against ARG-bearing bacteria is less common, related studies in biochar/hydrochar research suggest that adsorption and inactivation of pathogens often coincide with reduced bacterial culturability, implying damage to cell structure. Likewise, in composting with hydrochar, pathogenic (ARG-hosting) bacteria decline, which likely involves both chemical toxicity and membrane damage (or disruption of cell function; Shan et al., 2023). The direct physical and chemical interaction between hydrochar particles and bacterial cells can lead to membrane disruption and cell lysis. The mechanism is two-fold, involving both abrasive physical damage and oxidative stress.

The irregular surface morphology and sharp edges of certain hydrochar particles can cause physical damage to the cell envelope upon direct contact, compromising membrane integrity (Gao et al., 2018). In addition, the surface functional groups on hydrochar, particularly persistent free radicals (PFRs) that can form during carbonization, have been implicated in inducing oxidative stress (Sánchez-Silva et al., 2025). These PFRs can transfer electrons to dissolved oxygen, generating reactive oxygen species (ROS) such as superoxide anions and hydroxyl radicals (Tao et al., 2020; Liang et al., 2024). An onslaught of ROS leads to lipid peroxidation, protein dysfunction, and nucleic acid damage, ultimately resulting in the rupture of the cell membrane and the release of intracellular contents, including chromosomal and plasmid-borne ARGs (Tong et al., 2022). This lytic effect has a dual consequence. While it effectively inactivates the bacterial host, it simultaneously releases a pool of intracellular ARGs (iARGs) into the environment, converting them into eARGs available for horizontal gene transfer. The net impact on ARG abundance is therefore complex and contingent on whether the rate of bacterial inactivation and ARG degradation outweighs the rate of ARG release and subsequent stabilization through adsorption onto the hydrochar itself (Zhang et al., 2020b).

### 4.2 Indirect effects on soil and microbial communities

Hydrochar can indirectly reduce antibiotic resistance genes (ARGs) in slurry-soil systems by modifying antibiotic availability, metal dynamics, and soil-microbial interactions. Owing to its surface chemistry, hydrochar effectively adsorbs and, in some cases, catalyzes the transformation of antibiotics in soil, thereby lowering the selective pressure that sustains ARGs. For example, hydrochar amendments have been shown to reduce oxytetracycline (OTC) concentrations in soil and limit plant uptake by altering soil physicochemical properties and microbial metabolism (Lang Q. et al., 2023). Unlike pyrochar produced via dry pyrolysis, hydrochar is generated through hydrothermal carbonization (HTC), a wet process that preserves oxygen containing functional groups such as hydroxyl, carboxyl, and phenolic moieties. These functional groups confer a high cation exchange capacity (CEC) and strong sorption affinity for polar organic contaminants, including many veterinary antibiotics (Danso-Boateng et al., 2015). As a result, hydrochar sequesters antibiotics within soil matrices, reducing their bioavailability to both plants and soil microorganisms and, consequently, diminishing ARG maintenance driven by horizontal gene transfer (HGT; Lang S. et al., 2023).

Beyond antibiotic sequestration, hydrochar influences ARG dynamics by altering the geochemical behavior of heavy metals, a key driver of ARG co-selection. Metals such as Cu, Zn, and Cd promote antibiotic resistance through co-resistance and co-regulation mechanisms, as metal and antibiotic resistance genes frequently co-occur on mobile genetic elements or share stress response pathways (Babeker and Chen, 2021; Liu et al., 2025). HTC typically concentrates and stabilizes metals in the solid hydrochar phase, reducing their water soluble and exchangeable fractions and thereby decreasing metal mobility and bioavailability (Danso-Boateng et al., 2015). This immobilization weakens metal driven co-selection, contributing to declines in both metal resistance genes (MRGs) and ARGs, which often co-vary on integrons and transposons (Liu et al., 2025). Hydrochar also reshapes soil microbial habitats by modifying physicochemical properties such as pH, CEC, porosity, waterholding capacity, and organic matter content (de Jager and Giani, 2021; Jafari Tarf et al., 2021; Han et al., 2022). These changes alter microbial niche structure, oxygen availability, and nutrient accessibility, leading to shifts in bacterial community composition. Because ARGs are unevenly distributed across phylogenetic groups, changes in dominant taxa, particularly within ARG rich phyla such as Proteobacteria and Actinobacteria directly affect ARG abundance and diversity (Lang Q. et al., 2023; Yan et al., 2024). Notably, hydrochar induced changes in soil pH and CEC have been identified as stronger predictors of ARG variation than residual antibiotic concentrations, highlighting the dominance of indirect, community mediated mechanisms (Lang Q. et al., 2023). These indirect pathways act synergistically rather than independently. Enhanced porosity and water retention can stimulate microbial degradation of labile antibiotics not strongly sorbed by hydrochar (Jafari Tarf et al., 2021), while metal immobilization reduces oxidative stress and the activation of stress response regulons that incidentally upregulate antibiotic efflux systems (Lang Q. et al., 2023; Liu et al., 2025). Collectively, these processes position hydrochar as a multifunctional soil amendment that suppresses ARG proliferation by acting simultaneously as a contaminant sorbent, metal stabilizer, and microbial habitat engineer (Table 3).

**Table 3:** Hydrochar effects on key soil physico-chemical parameters and links to impacts on microbial community.

Physicochemical change	Effect on microbial community	Impact on ARG-hosting taxa	Key evidence
Alteration of pH	Can increase pH in acidic soils; generally, creates a more favorable environment for a broader range of microorganism (Vieira et al., 2026).	Indirectly suppresses acid-intolerant taxa that may carry ARGs.	Hydrochar from sewage sludge and thistle residues improved soil pH, which correlated with generally improved microbial biomass and activity (Di Santo et al., 2025).
Increase in cation exchange capacity (CEC)	Higher CEC improves nutrient retention and creates a more stable habitat, favoring the growth of a diverse and stable microbial community.	Suppresses allochthonous (non-native), opportunistic bacteria, including many potential pathogens.	A study showed that hydrochar addition, which can increase CEC, lowered the abundance of potential pathogenic bacteria during composting (Shan et al., 2023).
Increase in soil organic carbon (SOC)	Enhances microbial activity and shifts community composition toward slower-growing, more oligotrophic species that are efficient at decomposing complex carbon.	Suppresses specific phyla. For example, a decrease in the relative abundance of Actinobacteriota was linked to SOC accumulation and a reduction in carbon degradation functions.	Hydrochar addition decreased labile SOC fractions but increased stable SOC fractions, which was correlated with a reduction in *Sphingobacterium* (a labile carbon decomposer) and an increase in *Flavobacterium* and *Anaerolinea* (efficient decomposers of labile SOC; Sun et al., 2020).

Future research should prioritize long term field studies integrating metagenomics and ecological network analyses to disentangle these indirect mechanisms from direct antibiotic removal across multiple cropping cycles.

### 4.3 Influence of metals: HGT and bacterial phages

There are other anthropogenic, geogenic or naturally occurring factors that can also affect AMR in soils. Potentially toxic elements (PTEs) or “heavy metals” in soils can influence ARGs and therefore the levels of PTEs in soils need to be quantified if it can be considered as an additional stressor. PTEs often occur naturally in soils due to soil formation mechanisms such as chemical and physical weathering of the underlying bedrock, but they can also be deposited in soils through point source pollution events such as spills, burying of contaminated materials and through non-point pollution such as atmospheric deposition.

To assess the amount of PTEs in soils across a wide area, a background concentration must first be calculated in order to assess the baseline geochemistry (McIlwaine et al., 2014). This is the typical range of similar geochemical values that can be expected to be found within a population often due to a common controlling factor. Where the controlling factor can be attributed (such as the underlying geological unit, mineralization or pollution), then spatial “domains” can be defined where background concentrations can be calculated (McIlwaine et al., 2017). These background concentrations allow a direct comparison of study sites post the application of fertilizers/ soil amendments and can indicate if PTEs are there in elevated quantities that could cause stress on microbial communities.

The primary mechanisms by which PTEs influence ARG in soils are co-selection, microbial community shifts, and horizontal gene transfer. Co-resistance can occur due to some bacteria possessing genes for resistant phenotypes for both PTEs and antibiotics on the same plasmids (Baker-Austin et al., 2006). When there are elevated quantities (either background or added) of PTEs that can cause selective pressures, bacteria that develop resistance to PTEs on plasmids and proliferate, also indirectly increase antibiotic resistance. Other resistance mechanisms such as efflux pumps that transport PTEs and other compounds out of the cell (Gaurav et al., 2023), and the development of siderophores (Khan et al., 2024) can also protect bacteria from PTEs and antibiotics.

Hydrochars are normally low in PTEs which make them likely candidates as soil amendments (de Oliveira et al., 2026). The hydrothermal carbonization process can inhibit horizontal gene transfer by lowering the bio-availability of PTEs such Copper and Zinc (Chi et al., 2025) as well eliminating ARGs and mobile genetic elements. Wang and colleagues found agricultural hydrochars are efficient and removing PTEs such as Lead due to co-ordination complexation of –COOH and –NH_2_ groups (Wang et al., 2025). This suggests that hydrochar application can reduce PTE bioavailability and associated risks of horizontal gene transfer in areas where there are high background levels of PTEs or in urban environments where soils are more prone to contamination (Vienne et al., 2026).

## 5 Hydrochar application and subsequent effects on grassland soils

Studies have shown that hydrochar application can alter the microbial community structures, leading to a reduction in the abundance of pathogens and ARG host bacteria (Sun et al., 2020; Lang Q. et al., 2023; Shan et al., 2023). They also reported that hydrochar influenced microbial community succession during composting by modifying physico-chemical parameters, which aligned with the finding that environmental factors influenced host microorganisms and indirectly the ARGs. Moreover, acidic hydrochar can lower the soil pH from neutral and slightly acidic, increasing fungal richness and diversity while decreasing bacterial richness and diversity, suggesting an indirect effect through pH change (Sun et al., 2020). While the studies related to complex interactions between hydrochar and soil microbiota are under development, some researchers have reported a positive interaction between hydrochar and beneficial soil bacterial communities like *Proteobacteria, Firmicutes, Pseudomonas, Microbacterium*. The porous carbon matrix of hydrochar act as a habitat for these microbial population and acts as a slow release nutrient fertilizer contributing to the overall soil health (Armanu et al., 2025; Zhong et al., 2025; Sun et al., 2026).

A key study on hydrochar-derived organic fertilizers reported that amendment with hydrochar from beet chips led to divergent microbial responses, specifically increasing fungal diversity while reducing bacterial diversity (Gao et al., 2026). This pattern appears robust across different hydrochar feedstocks and application rates. The stimulation of fungal diversity presents a more complex mechanistic picture. Fungi generally exhibit broader pH tolerance than bacteria, allowing them to thrive under the acidic conditions that suppress bacterial competitors. The labile carbon fractions released from hydrochar, particularly dissolved organic carbon (DOC) may preferentially select for fungal taxa capable of metabolizing complex aromatic compounds. Furthermore, hydrochar's porous structure provides physical refugia for fungal hyphae while potentially excluding smaller bacterial cells from certain microhabitats. Fungi are primary decomposers of recalcitrant organic materials, including lignin and cellulose, due to their ability to produce extracellular oxidative enzymes such as peroxidases and laccases. The hydrochar-induced decrease in bacterial carbon degradation functions, coupled with increased fungal diversity, suggests a fundamental restructuring of decomposition pathways (Yao et al., 2025). This restructuring has dual consequences for soil carbon dynamics. On one hand, enhanced fungal decomposition may accelerate turnover of specific organic fractions. On the other hand, hydrochar amendment has been associated with increased soil organic carbon (SOC) accumulation, particularly particulate organic carbon (POC). This apparent paradox may be resolved by recognizing that fungal necromass contributes disproportionately to stable soil organic matter formation compared to bacterial biomass, owing to fungal cell wall components (chitin, melanin) that are inherently more recalcitrant.

Hydrochar application can mitigate the diffusion of ARGs when used as a soil amendment by lowering their enrichment and spread (Chi et al., 2025). Hydrochar may decrease the horizontal gene transfer capacity of HGT carriers by adsorbing them, which is one potential mechanism for reducing ARGs and mobile genetic elements (Shan et al., 2023).

Slurry storage and application is widely practiced in grassland animal agriculture systems and were known to have agronomic benefits, increasing nutrient availability and herbage yield (Lalor and Schulte, 2008; Köninger et al., 2021), in addition to providing a convenient farm waste management strategy. However, these benefits come with other environmental issues such as ammonia and greenhouse gas emissions, heavy metal deposition causing air quality issues and fastening climate change, nutrient leaching causing water pollution, etc. (Köninger et al., 2021; Baral et al., 2025). One of the overlooked issues was pathogen and AMR evolution from the practice of slurry application. Many studies have reported increase in heavy metal accumulation in soils with increased slurry applications (Zhou et al., 2016; Peng et al., 2022; Tan et al., 2024), and other studies have shown strong correlation between heavy metal and AMR evolution (Zhou et al., 2016; Mazhar et al., 2021; Tan et al., 2024; Balta et al., 2025; Kumar et al., 2025). Thus, despite agronomic benefits slurry applications bring a set of many other One Health issues. It is crucial that these issues from slurry are mitigated. Hydrochar could be one of the potential solutions.

Generally, hydrochar is known to increase nutrient availability in soils (Kwapinska et al., 2023), which in the absence of uptake by plants is highly prone to leaching. This increased nutrient availability is beneficial in some global regions where there is deficiency of macronutrients particularly phosphorus (P) and nitrogen (N), but may aggravate the eutrophication problem in some regions with nutrient-rich soils. Nevertheless, there are studies indicating some of the phosphorus fractions become less available after hydrochar treatment (Huang and Tang, 2015, 2016), which may potentially reduce phosphorus leaching issues with slurry application. Studies on the impact of different hydrochar and their impact on application onto nutrient rich soils to clearly understand the mechanisms and impacts, are needed. However, hydrochar application can also result in improved soil porosity and structure, aiding soils to store more water and avoid leaching loss by retaining nutrients for a longer time in the soil for the plants to use. This could potentially mitigate nutrient pollution issues caused by slurry application. The mechanism of hydrochar slurry application needs to be considered as it influences soil microbiota. For instance, slurry subsurface injection methods have been proven to reduce the ammonia emissions by altering the spatial nutrient distribution, molecular oxygen bioavailability and physical microenvironment (Pintarič et al., 2022; Thorn et al., 2025). In addition, adsorption properties associated with hydrochar can also help mitigate heavy metals and pollutants in soils from slurry application (Fakkaew et al., 2018), further mitigating AMR/ARG development in grassland soils.

Soils have natural resistomes in their native microbiome that helps in disease prevention by mitigating introduced pathogens (Zhu et al., 2019), in fact Banerjee and van der Heijden (2022) lists 40 different functions of soil microbiome that directly or indirectly relates to One Health. Any alterations in microbiome due to long term grassland management such as excessive nutrient application can result in changes in microbiomes and impact these One Health services provided by the soil microbiome. Heavy metal and antibiotics accumulation in soil are characteristically associated with slurry application in grasslands which were known to significantly alter soil microbiome and greatly impact these services. Hydrochar treatment of slurry can help mitigate these impacts by reducing the impact on native microbiome by controlling the accumulation of heavy metals and antibiotics. Slurry application also causes complexity in nutrient management as they are highly heterogenous and hence results in uneven spread of nutrients across the grassland field (Higgins et al., 2019). This causes difficulties in agronomic nutrient management and in controlling nutrient leaching. Hydrochar treated slurry which is a solid material, could increase the consistency of mineral content and help with efficient nutrient management that can benefit both agronomic production and environmental protection.

Another key One Health issue associated with slurry storage and applications is ammonia and greenhouse gas (GHG) emissions (Baral et al., 2025). Ammonia emissions can occur both during storage and application of slurry in grasslands, and have potential to cause human and ecosystem health issues (Guthrie et al., 2018). Sensitive, nutrient poor and carbon rich habitats like peatlands are highly prone to be affected by ammonia deposition (Guthrie et al., 2018). Degradation of peatland and forest ecosystems by ammonia deposition impacts the valuable ecosystem services provided by these natural habitats, significantly impacting planetary health (Guthrie et al., 2018; Gurmesa et al., 2022). Current mitigation strategies for ammonia emissions during slurry storage involve acidification of slurry, using oxidizers, or floating covers (Kupper et al., 2020; Langley-Randall et al., 2024). Different low emission technologies are used and nationally regulated in most countries to reduce ammonia emissions during slurry applications (Huijsmans et al., 2016). Injection method for slurry application has generally been most efficient method for ammonia emission reduction during slurry application; however, it is not the most convenient method. Hydrochar with its pH reducing properties can help reduce ammonia emissions both during storage and application to grasslands while retaining all the agronomic benefits associated with untreated slurry application. However, the practical and economic feasibility of hydrochar production from slurry at scale needs to be researched.

Slurry application to soil is known to improve soil carbon sequestration (Fornara et al., 2016) with minimal impact on soil to atmosphere carbon emissions (Holland et al., 2024). Hydrochar treatments would enhance the carbon sequestration potential due to further stabilization of carbon through hydrothermal carbonization process (Bever and Coronella, 2024; Luo et al., 2025). Bever and Coronella (2024) found that hydrochar from manure substantially increased the carbon sequestration potential, however not as much as biochar produced from the same manure. However, production of biochar requires solid feedstock, which makes hydrochar a suitable option to treat slurry that has high liquid content, to enhance carbon sequestration. Nevertheless, there is further research needed on the impact of slurry properties on hydrochar and their potential benefits. Overall, in addition to AMR/ARG mitigation, hydrochar shows high potential in mitigating other environmental issues and enhancing ecosystem services from slurry application to grassland soils.

## 6 Environmental and economic considerations

A cradle-to-cradle sustainability assessment of hydrochar production on farms needs to be linked with an integrated life cycle assessment and material management approach. This should consider feedstock supply chains, material handling and storage, process energy demand, carbon counting, and any future circular economy valorization of hydrochar products. Additional societal and economic benefits from the prevention AMR in agricultural soils should be considered. The availability of farm slurry and the logistics of processing (either on farm or through a local hub site) are key factors in the success of such an approach. Hydrothermal processing of hydrochar is a versatile method that can handle a variety of slurry material and agri-wastes but the scalability of a sustainable approach can depend on the availability of reliable, homogenous input material, the amount of pre / post processing, and minimized transport distances to reduce environmental impact (Ogunleye et al., 2024). Good design of the hydrochar manufacture and material management is essential to manage energy consumption during the hydrochar production process. Energy consumption can dominate the environmental footprint (Mohammadi et al., 2020; Yin et al., 2023). The energy inputs for heating and drying of materials are significant factors and that efficient energy recovery of waste heat and renewables can improve performance (Glover et al., 2023). It has been recognized that hydrochars and biochars can reduce short-term soil CO_2_ and N_2_O fluxes when correctly applied, resulting in reduction in global warming potential (Joshi et al., 2022) but care must be taken to ensure that process design, feedstock type, hydrochar properties, and the chosen valorization route of the hydrochar allow for sustainable outcomes. The Life-cycle Assessment of char production and application in soil is a different comprehensive study by itself as it involves variable production parameters, complex stoichiometric mass balance, analytical dataset and inventories. To give an overview, a study on the LCA of biochar and hydrochar production revealed that the former had a Global Warming Potential (GWP) of around −11.7–9.1 kg CO_2_e while the latter had a GWP of around −71.4–7.7 kg CO_2_e with lower environmental footprint (Gievers et al., 2025).

## 7 Knowledge gaps, future research directions and conclusion

While hydrothermal carbonization (HTC) effectively reduces antibiotic resistance genes (ARGs) in raw slurry, its application to grassland soils as a One Health solution introduces significant unresolved complexities. Current knowledge is limited by short-term studies, a focus on bulk soil, and a lack of mechanistic understanding of post-application dynamics. Most studies quantify total ARG abundance, ignoring the mechanistic distinction between intracellular (iARGs) and extracellular (eARGs) genetic material. Future research must determine whether hydrochar adsorbs and preserves eARGs, facilitating horizontal gene transfer (HGT) via transformation in grassland soils, which are subject to frequent wet-dry cycles.

Moreover, hydrochar can retain concentrated heavy metals (e.g., Cu, Zn). A critical gap is understanding how these residual constituents exert co-selective pressure on the soil resistome. Future studies focused on metagenomic analyses linking ARGs with metal resistance genes (MRGs) and mobile genetic elements (MGEs) are required to differentiate direct effects from co-selection. In addition, grassland soils are structured by macroaggregates, root channels, and earthworm activity. Research must move beyond bulk soil analysis to investigate ARG dynamics in micro-niches such as the rhizosphere, drilosphere (earthworm-affected zones), and leachate. Specifically, the role of endogeic earthworms in translocating hydrochar-borne contaminants and ARGs from the surface to deeper soil layers remains unexplored.

Furthermore, a significant gap is the potential for hydrochar to alter the endophytic resistome of pasture grasses. Future studies should track ARG transfer from hydrochar-amended soil to the phyllosphere (leaf surface) and internal plant tissues, assessing whether grazing livestock act as a direct pathway for resistance genes to reach the food chain. Also, existing evidence is derived almost exclusively from short-term incubations (weeks to months). Multi-year field trials are essential to capture chronic effects, including the impact of repeated hydrochar applications, seasonal soil freeze-thaw cycles, and the long-term resilience of the soil resistome. Key questions include whether initial ARG suppression is sustained or followed by a late stage rebound due to shifts in microbial community structure.

A lack of standardized HTC parameters (e.g., temperature, residence time) and application rates hinders cross-study comparison. Future studies must define the processing conditions that maximize ARG destruction in slurry while minimizing the formation of co-selecting agents and preserving the agronomic value of the hydrochar for grassland systems. Finally, there is an urgent need for holistic evaluations that couple ARG mitigation efficacy with life cycle assessment (LCA). Research should integrate environmental (soil health, water quality), agronomic (grass yield, soil carbon), and public health (ARG exposure risk) endpoints to present a holistic view on the role slurry-derived hydrochar as a genuine One Health solution in the terrestrial environment.

Future research should also focus on the production of hydrochar from slurry before application to grasslands, providing a novel and practical solution to mitigate ARGs, thereby enhancing One Health, and potentially enhancing grassland soil nutrient health. In addition, more field-scale studies to assess the performance of hydrochar-amended slurry under realistic environmental conditions and over extended periods are needed as long-term monitoring will be vital in evaluating ARG persistence and potential rebound. Overall, we identified the potential of hydrochar as a valuable tool for reducing ARG dissemination from slurry in grassland soils and promoting sustainable agricultural practices.
